# Precise Control over the Spatial Arrangement of Copper Selenide on Au Nanobipyramids by Site-Selective Growth for Dual Plasmonic Nanoarchitectures

**DOI:** 10.1021/acsnanoscienceau.5c00102

**Published:** 2025-10-20

**Authors:** Au Lac Nguyen, Hao Jing

**Affiliations:** Department of Chemistry and Biochemistry, 3298 George Mason University, Fairfax, Virginia 22030, United States

**Keywords:** Gold nano bipyramids, Nonstoichiometric copper chalcogenides, Dual plasmonic heterostructures, Spatial arrangement, Regioselective overgrowth, Surfactants

## Abstract

Dual plasmonic heterostructures composed of gold nanoparticles (Au NPs) and nonstoichiometric copper chalcogenides (Cu_2‑x_E) have garnered attention for their unique electronic interactions between two intrinsically dissimilar constituent domains. However, the site-selective deposition of Cu_2‑x_E on Au NPs remains extremely challenging due to the difficulty in controlling nucleation and regioselective overgrowth. Herein, we propose a universal Selenide (Se)-mediated approach for precise spatial control of Cu_2‑x_Se on gold nano bipyramids (Au NBPs). By deliberately tuning the surfactant environment, Cu_2‑x_Se can be selectively deposited on one waist, both lateral sides, and tips of Au NBPs to form UFO-like, segregated islands, and spindle-like morphologies, respectively. Furthermore, the domain size of the Cu_2‑x_Se and the plasmonic properties of Au@Cu_2‑x_Se can be controlled by adjusting the amount of selenium (SeO_2_) precursor. This work establishes a new strategy for the rational design and fabrication of multicomponent functional nanoarchitectures with precisely controlled compositions and tailored plasmonic properties, thereby expanding their scope of applications.

## Graphical abstract

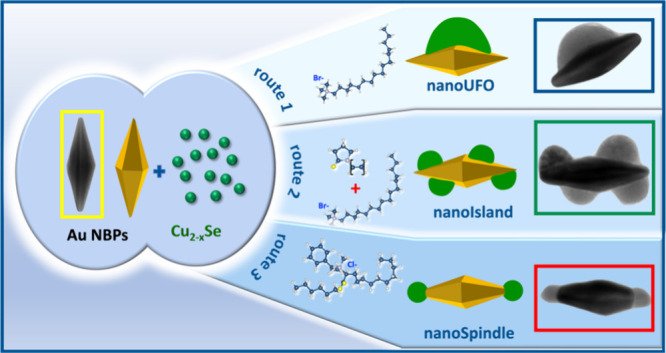

## Background

Dual plasmonic heteronanostructures composed of noble metals and semiconductors are receiving increasing scientific attention, partly due to their intriguing optical properties arising from plasmonic coupling.^1–5^ Particularly, heterostructures comprising Au NPs and vacancy-doped copper chalcogenides (Cu_2‑x_E, *x* > 0, E = S, Se, Te) are known for harnessing the phenomenon of the attractive localized surface plasmon resonance (LSPR) and possess great potential for enhancing the light-driven chemical processes.^6–8^ They have served as the foundation for multifunctional materials in plasmon-based applications such as photocatalysts,^9^ photoelectrocatalysts,^10^ nanoantenna for H_2_ evolution,^11^ electrocatalytic N_2_ reduction,^10^ deep tissue imaging,^8^ photosynthesis of nitrate from air,^12^ and chemophototherapy of cancer.^13,14^

Among Cu_2‑x_E compounds, Cu_2‑x_Se is widely used for regioselective doping onto the Au NPs as the sacrificial template to construct dual plasmonic hetero nanostructures based on two key factors: 1) its LSPR band is tunable through controlling the degree of copper deficiency via oxidation upon exposure to oxygen;^15–17^ and 2) The plasmonic properties of Cu_2–x_Se arise from copper vacancies (Cu_2–x_Se, *x* > 0), which function as intrinsic dopants, generating a strongly p-type semiconductor. Each Cu vacancy introduces a free hole into the valence band, and at sufficiently high vacancy concentrations, the hole density can reach ∼ 10^21^ cm^–3^. This high carrier density enables collective oscillations of holes under light irradiation, observed as LSPR. Owing to the larger effective mass of holes relative to electrons in metals, LSPR occurs in the NIR. Crucially, both the resonance wavelength and intensity of this resonance can be precisely tuned by controlling the copper deficiency, making Cu_2–x_Se a stoichiometry-dependent plasmonic semiconductor.^18^ Integrating plasmonic Au NPs with Cu_2–x_Se into a single entity creates a dual-plasmonic hybrid that greatly enhances a range of photophysical and photochemical processes. These enhancements depend critically on the size, shape, and uniformity of the Au NPs, the Cu_2–x_Se domain size, and the deliberate engineering of the Au@Cu_2‑x_Se architecture.^18–21^ Notably, the dual plasmonic nanostructures composed of Au NPs doped with copper chalcogenide exhibit a stronger local electric field enhancement at the interface than the sole excitation of pristine Au NPs or Cu_2‑x_E under excited LSPRs, which leads to a dramatic enhancement of photocatalytic properties.^22,23^

Among the diverse morphologies of plasmonic Au NPs, Au NBPs are particularly attractive as nanoplatforms for forming hetero nanoarchitectures due to two key advantages. First, their sharp dual tips induce a tunable longitudinal LSPR, a high molar extinction coefficient, and local electric field enhancements approximately 5-fold greater than those observed at the ends of Au NRs with the same aspect ratio.^24–27^ This pronounced LSPR leads to higher production rates of hot electrons.^28,29^ Moreover, the Au NBPs possess larger optical cross sections, narrower line widths, and higher refractive index sensitivity than Au NRs.^25,30^ Therefore, the rational design of site-selectively Cu_2‑x_Se on the surface of Au NBPs, forming a dual plasmonic heteronanostructure, can maximize hot carrier generation/harvesting and remarkably promote charge separation.^31^ However, to the best of our knowledge, achieving such precise control on a single nanostructure remains a significant challenge and has not yet been thoroughly explored to date.

In this work, we report the precise control of the spatial arrangement of plasmonic semiconductors (nonstoichiometric copper chalcogenides) on plasmonic noble metal nanostructures for the first time. It can be achieved by a general route for the site-selective deposition of Cu_2‑x_Se on various positions of anisotropic Au NBPs’ surfaces to form different morphologies, including nanoUFO, nanoisland, and nanospindle heteronanostructures. The Cu_2‑x_Se domain is synthesized by Se-mediated approaches and selectively deposited at one waist side, on both lateral sides, and on both tips of Au NBPs, whose surfaces are modified by using a variety of surfactants such as hexadecyltrimethylammonium bromide (CTAB), CTAB and polyvinylpyrrolidone (PVP) mixture, and Benzyl dimethyl hexadecyl ammonium chloride (BDAC), to form nanoUFO, nanoisland, and nanospindle configurations, respectively. Our approach not only precisely designs the site-selective overgrowth position of Cu_2‑x_Se on the surface of Au NBPs, but it can also be tuned to the domain size of Cu_2‑x_Se by adjusting the amount of SeO_2_ precursor. This can allow for tuning the plasmonic optical properties of Au@Cu_2‑x_Se, thereby broadening its applications. More importantly, A deeper understanding of the underlying mechanism in the overgrowth patterns could offer valuable insights and enriched perspectives for future research on several fundamental and open questions, including how to facilitate the charge carrier migration across the interface between constituent domains, what are the optimal nanostructures to elucidate cross-interactions between two mechanistically distinct LSPRs with ultrafast dynamics, and how to efficiently utilize hot-electrons and/or hot-holes in plasmonic mediated) photocatalysis.^32–35^

## Results and Discussion

High-yield Au NBPs were synthesized via a seed-mediated method by tuning the binary surfactant at the seed step, as described in our previous work.^27^ As-prepared Au NBPs exhibit highly uniform shape and a narrow size distribution, with average dimensions of 112 ± 4.4 (length) and 42.2 ± 3.1 nm (width), as shown in Figure S1A, C, and D. UV–vis-NIR spectra further confirm a high purity of Au NBPs, with a pronounced LSPR band around 800 nm. The weaker peak around 550 nm is attributed to the transversal mode of the LSPR, and is contributed from a small amount of shape impurities (Figure S1B).^36^ This high-quality Au NBPs platform enabled regioselective overgrowth of Cu_2‑x_Se, yielding three distinct constructions through controlled patterning.

Herein, we developed three efficient routes for site-selective overgrowth of Cu_2‑x_Se on anisotropic Au NBPs (Au@Cu_2‑x_Se), enabling precise morphological control through a two-step Se-mediated process. In the first step, amorphous Se is site-selectively deposited onto the capping agent-stabilized Au NBPs surface by reducing the SeO_2_ with Ascorbic acid (AA) to form Au@Se. This step primarily governed the morphology of Au@Cu_2‑x_Se, as the preferential overgrowth site of elemental Se is dictated by the surface-bound capping agents stabilized on the Au NBPs.^37^ Figure 1 depicts the growth behavior of Se on Au NBPs stabilized by various surfactants, allowing site-specific nucleation and overgrowth of Cu_2‑x_Se while passivating other facets, thereby yielding various heteronanostructure morphologies.^20^ In the second step, the simultaneous addition of a Cu^2+^ precursor and AA leads to the reduction of Cu^2+^ to Cu^+^, which then reacts with the Se template to form Cu_2_Se on the site-selective surface of Au NBPs. Cu_2_Se was then oxidized to Cu_2‑x_Se upon exposure to air.^15,20,22^

**Figure 1:**
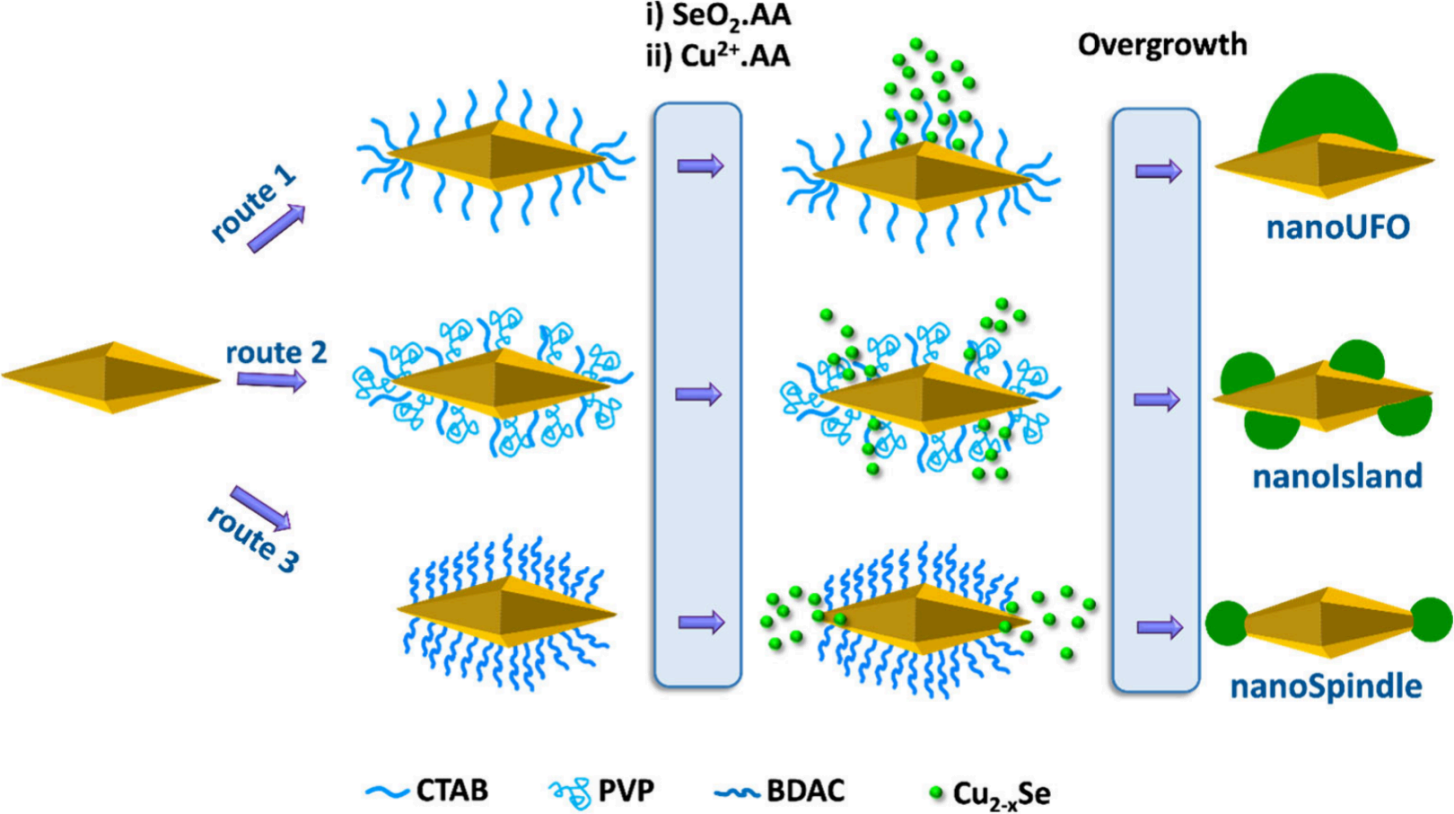
Schematic illustration of the site-selective growth of Cu_2‑x_Se on the Au NBP with various surfactants to form different Au@Cu_2‑x_Se morphologies.

Figure 2 illustrates the site-selective growth of Cu_2‑x_Se on distinct regions of the Au NBPs surface, directed by different capping agents, including CTAB, CTAB-PVP mixture, and BDAC. CTAB is a bromide-containing cationic surfactant composed of quaternary ammonium positive charges and bromide ions.^38,39^ At appropriate concentrations, when a CTAB bilayer stabilized Au NBPs, Cu_2‑x_Se mainly overgrew on one side of the Au NBPs due to initial site-selective nucleation. Nucleation requires overcoming a local energy barrier; thus, the regions of the Au NBPs that are less densely passivated by CTAB serve as preferential nucleation sites for the Se precursor. These sites become kinetically favored for subsequent growth. Once nucleation is initiated at a single side, kinetic factors combined with local electric field enhancement promote continued deposition at that location rather than across the entire particle.^34,40^ As a result, Cu_2‑x_Se is solely embedded onto the waist of CTAB-stabilized Au NBPs and mainly grows on one side of the Au NBPs’ waist (Figure 2A). That makes the Au@Cu_2‑x_Se heteronanostructure look like a nanoUFO. Furthermore, the extinction UV–vis-NIR spectra of nanoUFO shows that the longitudinal LSPR did not shift compared to the as-prepared Au NBPs, as shown in Figure 2D. Specifically, there is no tip overgrowth, resulting in no enhanced plasmonic coupling of the longitudinal LSPR in the nanoUFO sample.

**Figure 2:**
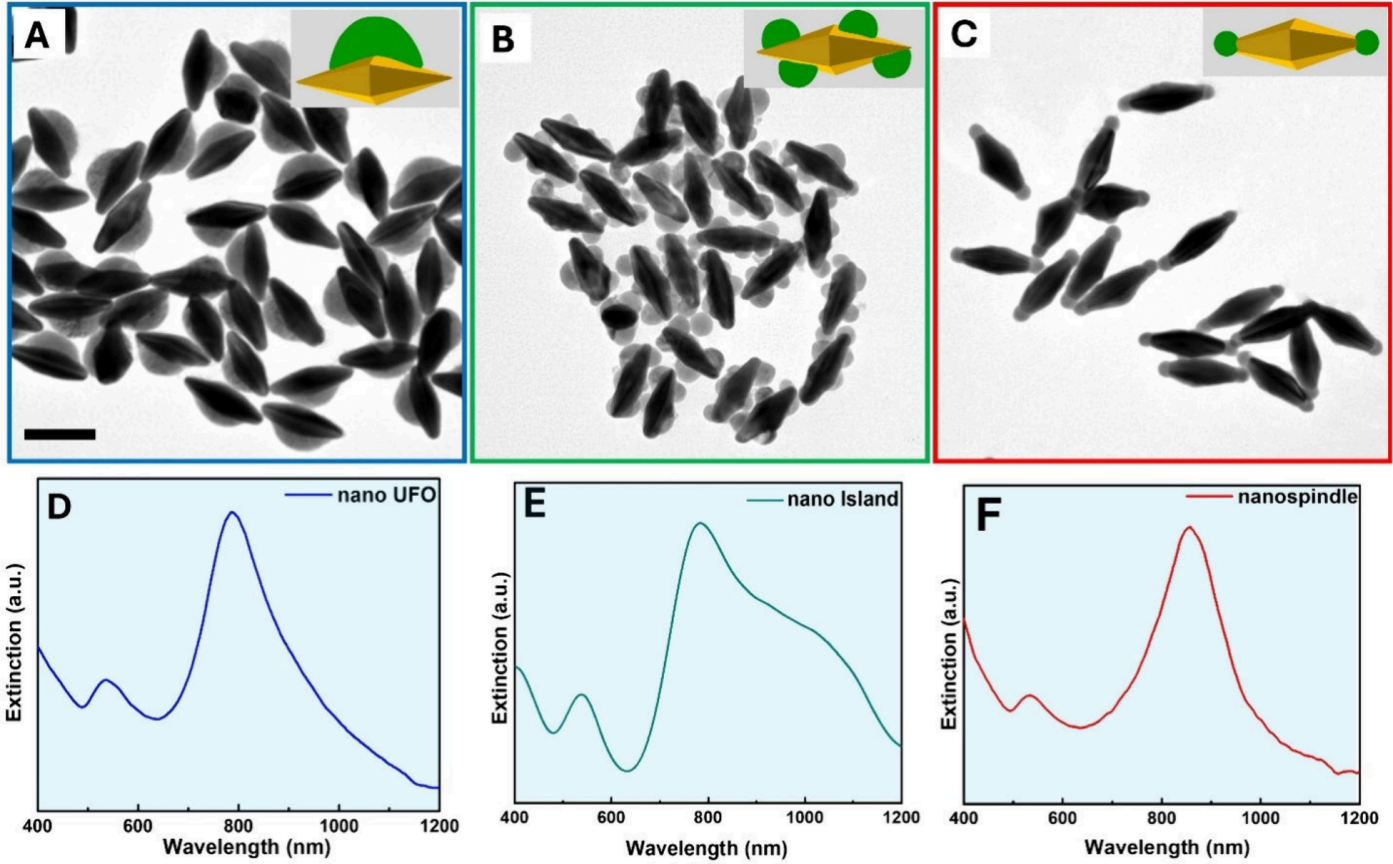
Site-selective deposition of Cu_2‑x_Se on Au NBPs. (A), (B), (C) TEM images of Au @Cu_2‑x_Se nano UFO, Au@Cu_2‑x_Se nanoisland, and Au@Cu_2‑x_Se nanospindle, respectively. (D), (E), (F) Extinction spectra of Au@Cu_2‑x_Se nanoUFO, nanoisland, and nanospindle, respectively. The scale bar is 100 nm.

When the binary mixture of CTAB and PVP was utilized to stabilize the surface of Au NBPs, the Cu_2‑x_Se was selectively overgrown on the different lateral sides of the CTAB-PVP mixture-stabilized Au NBPs instead of solely one side of the CTAB-stabilized Au NBP’s belly. This led to the formation of uniform and monodispersed Au@Cu_2‑x_Se with multiple smaller Cu_2‑x_Se islands distributed on the lateral sides, as illustrated in the TEM images (Figure 2B). The site-selective overgrowth of Cu_2‑x_Se on the sides of the CTAB-PVP mixture-stabilized Au NBPs can be explained bromide ions (Br^–^) from CTAB preferentially adsorb onto the {100} facets of Au, modulating surface energy and suppressing growth along these directions. besides that, in the presence of PVP, the carbonyl oxygen in the pyrrolidone ring of PVP exhibits a strong dipole that engages in ion–dipole interactions with the bromide-modified surface, while the nitrogen atom contributes indirectly by stabilizing the dipole orientation. This cooperative adsorption stabilizes specific crystal facets more effectively than Br^–^ or PVP alone, thereby directing the nucleation and formation of multiple small Cu_2‑x_Se domains on the lateral surfaces of CTAB–PVP-stabilized Au NBPs. This prevents the deposition of Cu_2‑x_Se along the Au [111] surface and large curvature. As a result, Cu_2‑x_Se site-selectively deposited on the lateral surfaces of the CTAB-PVP mixture-stabilized Au NBPs.^41–43^ To examine the optical properties of this heterostructure, the extinction UV–vis-NIR spectra were obtained as shown in Figure 2E. A broad, red-shifted shoulder was observed near the main LSPR band, attributed to a combination of electronic interaction and plasmonic coupling between Au NBPs and Cu_2‑x_Se, particularly in the tip regions.

Notably, Au@Cu_2‑x_Se spindle-like heterostructure was successfully synthesized with high-quality and high-yield preparation of Au@TIP-Cu_2‑x_Se as depicted in Figure 2C. While the Au@Cu_2‑x_Se nanospindle was produced following a two-step procedure, its synthesis method differs from those of the nanoUFO and nanoisland procedures. Particularly, the surface of Au NBPs was carefully washed twice to remove the excess CTAB surfactant. The Au NBPs surface is then treated by using a low AgNO_3_ concentration in BDAC solution. In this step, Ag^+^ was used to serve as a sacrificial mediator to enable ligand exchange on Au nanocrystals. By first depositing an ultrathin Ag shell onto the Au NBP surface, the strong CTAB binding is disrupted, since Ag^+^ does not interact with CTAB as strongly as Au. and subsequent etching in the presence of BDAC allows BDAC molecules to occupy the newly exposed surface sites. This process effectively replaces CTAB with BDAC, yielding Au nanocrystals stabilized by BDAC surfactants while minimizing CTAB contamination, as demonstrated in Figure S2. The BDAC surfactant preferentially adsorbs on the lateral side of Au NBPs, but not as densely on their sharp tips. Hence, the tips have the lowest BDAC surfactant density and leave the tips less protected, which allows Se precursor nucleation selectively at the tips and forms the nano spindle.^44^ However, it is essential to note that the concentration of BDAC plays a crucial role in determining the selectivity and extent of Cu_2‑x_Se growth on Au NBPs. At a concentration of 1 mM, the BDAC molecules were insufficient to form a dense protective layer on the Au surface. As a result, the stabilization of the surfactant bilayer was incomplete, leaving both the side facets and the tips partially exposed. Under these conditions, Cu_2‑x_Se precursor ions could access multiple sites, leading to growth across the entire surface of the Au NBPs, as observed in Figure S3A. In contrast, when the BDAC concentration was increased to 10 mM, the Au NBPs were densely passivated by BDAC molecules, which effectively inhibited Cu_2‑x_Se deposition on both the tips and the side surfaces of the Au NBPs. Consequently, as shown in Figure S3B, a fraction of particles remained uncoated, while some displayed partial deposition restricted to a single tip. Interestingly, at a concentration of 5 mM BDAC, Cu_2‑x_Se was selectively deposited at the tip of BDAC-stabilized Au NBPs due to high curvature. Besides that, the low concentration of AA was used to slow down the reaction rate of SeO_2_, which can control all Se overgrowth at the tip of BDAC-Stabilized Au NBPs.^33,45^ Notably, the extinction UV–vis-NIR spectra of the nanospindle sample showed the characteristic of an extinction peak around 856 nm (Figure 2F), which exhibits the largest red shift compared to other previous samples. This is because the Sharp tips of Au NBPs concentrate high electric fields, which modulate LSPR signals with high sensitivity.^46^ Besides that, the optical bandgap energies of the Au@Cu_2–x_Se samples were estimated from UV–Vis absorption spectra (Figure 2D-F) using Tauc plots. The UFO, Island, and Spindle morphologies exhibited bandgap energies of 2.30, 2.32, and 2.34 eV, respectively (Figure S4). Additionally, Raman spectra also demonstrated the characterization of the surface-binding ligands of three Au@Cu_2‑x_Se hetero nanostructures functionalized with different surfactants (Figure S5).

To further investigate the spatial distribution of Cu_2‑x_Se on three morphologies of synthesized Au@Cu_2‑x_Se heteronanostructures, we employed high-angle annular dark-field scanning transmission electron microscopy (HAADF-STEM) imaging and energy-dispersive X-ray (EDX) elemental mapping. As shown in Figures 3–5, Au is consistently localized in the core of the heterostructures, while the distribution of Cu and Se varies depending on the morphology. In the nanoUFO-type structure, Cu and Se are precisely located at the belly. In contrast, the nanoisland exhibits that Cu and Se elements are found on the lateral surface of both sides of the Au NBPs. For the nanospindle-like, Cu and Se were deposited at the tips of Au NBPs. More intriguingly, Figure 5B revealed that submonolayer Ag atoms coated the surface of the nanospindle and combined with Au to form a surface alloy. This can dramatically inhibit Au atomic diffusion and greatly enhance the stability of the nanoheterostructure.^32^ Additionally, HR TEM Figure 5G revealed that Cu_2‑x_Se possessed high crystallinity with the interplanar spacings of 0.333 and 0.236 nm corresponding to the [111] facet of Cu_2‑x_Se and Au NBPs domain, respectively. Furthermore, X-ray diffraction (XRD, Figure S6) analysis was carried out to further investigate the structural characteristics of the Au@Cu_2–x_Se and Cu_2–x_Se samples. As shown in the diffraction patterns, the Au@Cu_2–x_Se heterostructure exhibits well-defined peaks, confirming its high crystallinity.

**Figure 3:**
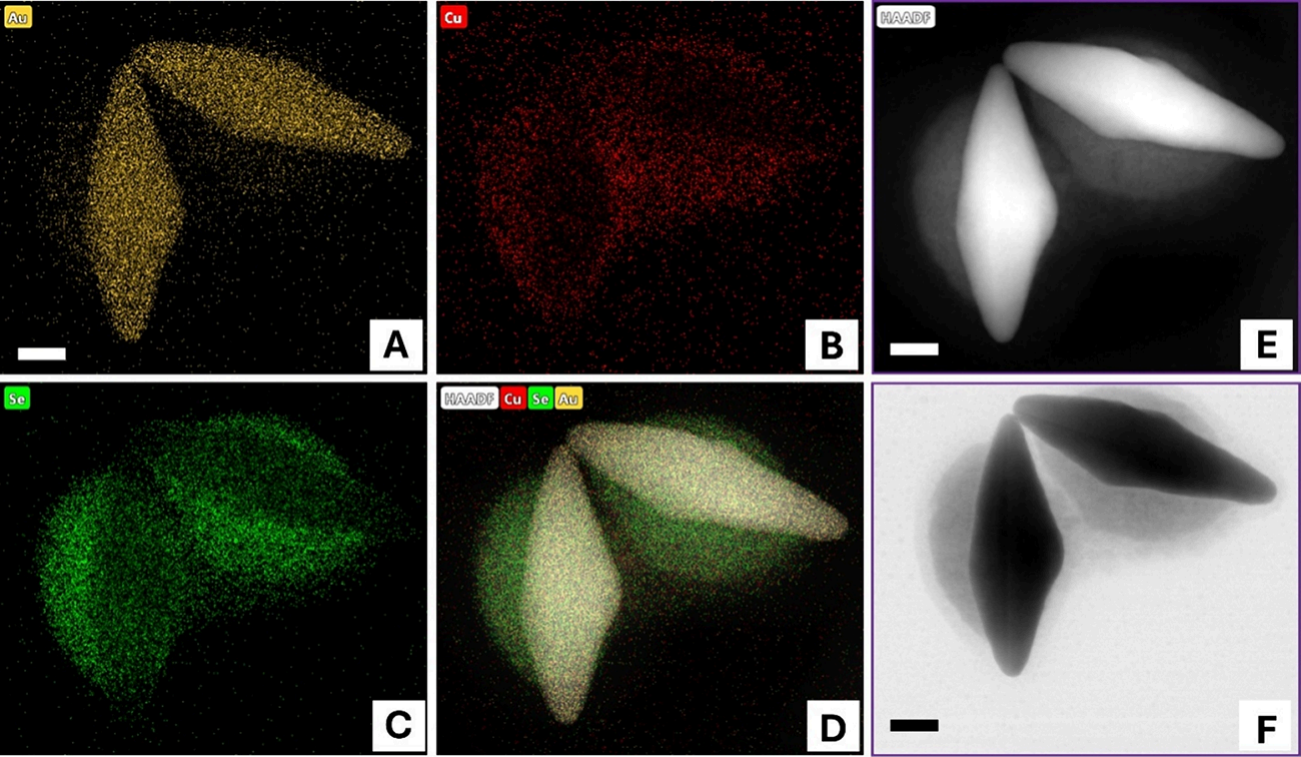
HR-TEM and high-magnification HAADF-STEM images and corresponding EDS mapping showing the distribution of Au, Cu, and Se on Au@Cu_2‑x_Se nanoUFO heteronanostructures. The scale bar is 20 nm.

**4:**
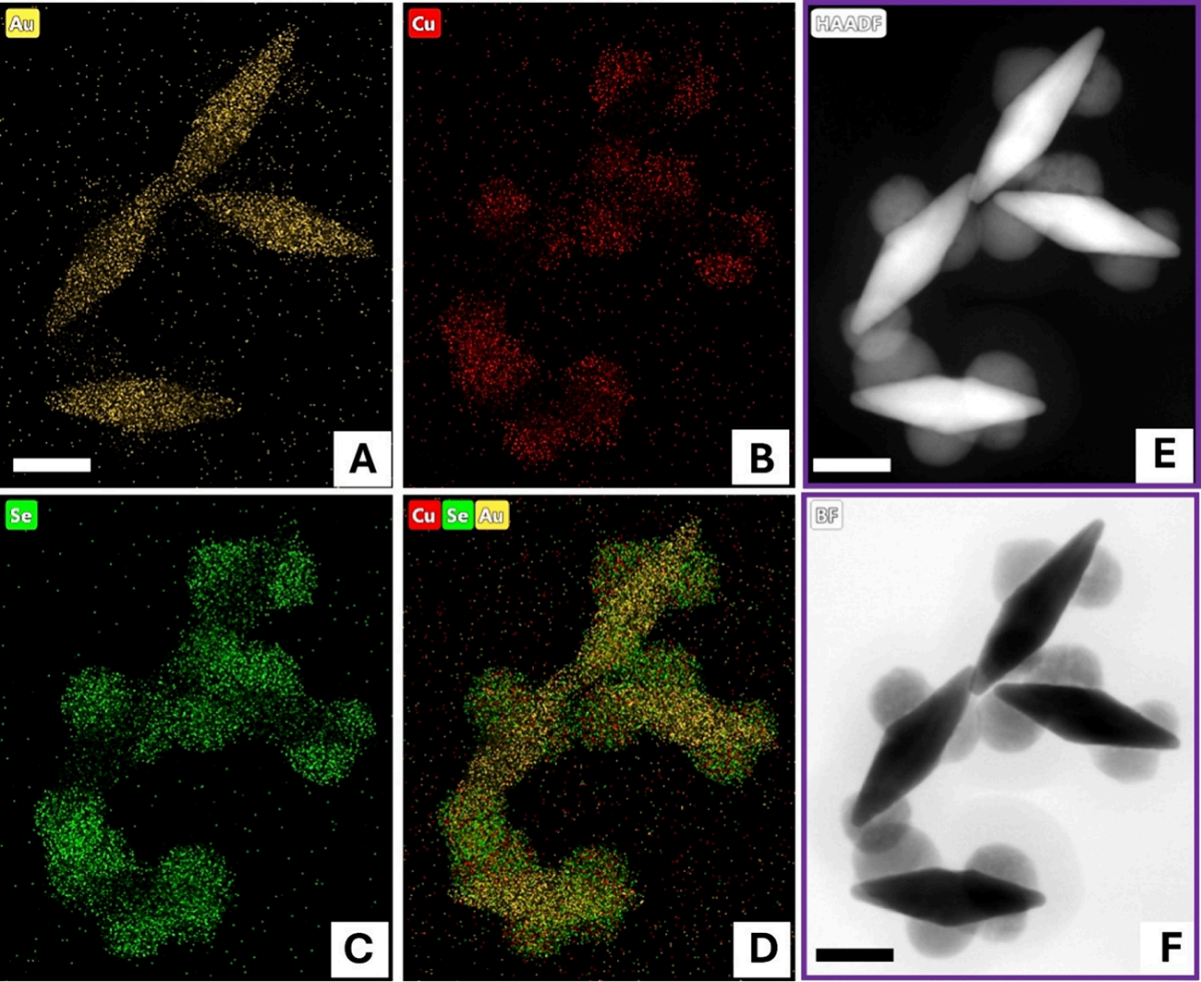
HR-TEM and high-magnification HAADF-STEM image and corresponding EDS mapping showing the distribution of Au, Cu, and Se on Au@Cu_2‑x_Se nanoisland heteronanostructures. The scale bar is 20 nm.

**Figure 5:**
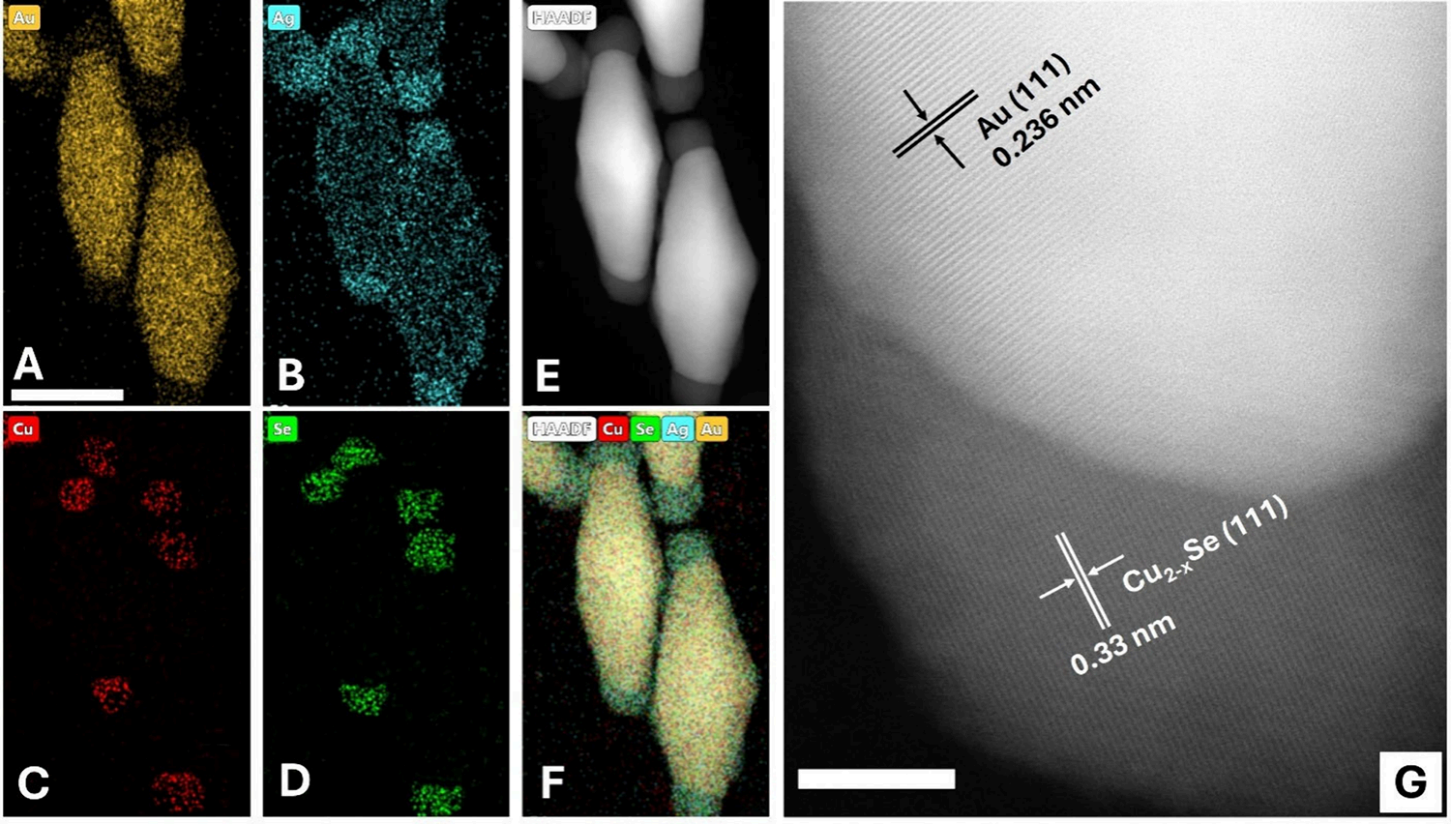
HR-TEM and high-magnification HAADF-STEM image and corresponding EDS mapping showing the distribution of Au, Ag, Cu, and Se on Au@Cu_2‑x_Se nanospindle heteronanostructures. The scale bars are 50 nm (A-F) and 5 nm (G).

Notably, Cu_2‑x_Se domain size on the Au@Cu_2‑x_Se nanoUFO and nanoisland can be controlled by adjusting the amount of SeO_2_ precursor (Figures 6A-C and Figures 6D-F). Specifically, Cu_2‑x_Se domain size was proportionally increased with the amount of Se addition, and its location depends on the structure type. While Cu_2‑x_Se was selectively located only on one side of the nanoUFO, the nanoisland sample experienced the growth of Cu_2‑x_Se on the lateral side, which is further confirmed in Figures S7–8. The extinction UV–vis-NIR spectra of nanoUFO also indicated that when a higher amount of 1 mM SeO_2_ was used, the LSPRs shifted to the redshift for both structure types. This can be explained by the fact that as Cu_2‑x_Se becomes larger, it approaches the sharp tip of the Au NBPs. This causes electronic interaction and plasmonic coupling between Au NBPs and Cu_2‑x_Se. However, LSPRs of nanoisland samples remained unchanged. The broader red-shifted shoulder was observed when a higher amount of SeO_2_ was used. This can be explained by the fact that as Cu_2‑x_Se becomes larger, it approaches the sharp tip of the Au NBPs.

**Figure 6:**
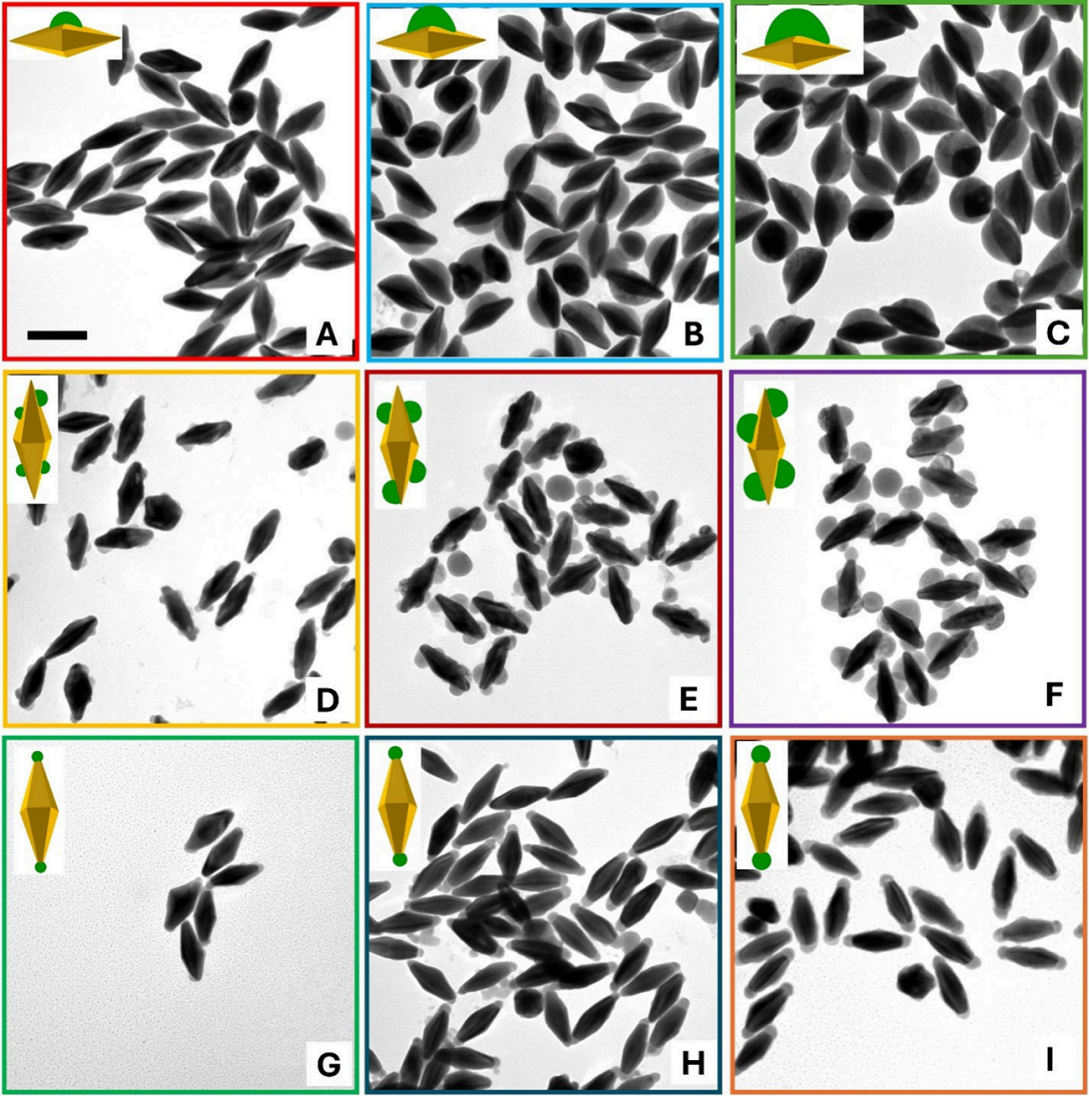
Site-selective deposition of Cu_2‑x_Se on Au NBPs with increasing Cu_2‑x_Se domain size. TEM images of Au@Cu_2‑x_Se nanoUFO heteronanostructure with increasing Cu_2‑x_Se size obtained using (A) 25 μL, (B) 50 μL, and (C) 100 μL of 1 mM SeO_2_. TEM images of Au@Cu_2‑x_Se nanoisland heteronanostructure with increasing Cu_2‑x_Se size obtained using (D) 25 μL, (E) 50 μL, and (F) 100 μL of 1 mM SeO_2_. TEM images of Au@Cu_2‑x_Se nanospindle heteronanostructure with increasing Cu_2‑x_Se size obtained using (G) 25 μL, (H) 50 μL, and (I) 75 μL of 1 mM SeO_2_, with different concentrations of BDAC: 3, 5, and 7 mM. The scale bar is 100 nm.

The representative TEM images of the as-obtained nanospindle structure with various lengths of Cu_2‑x_Se deposition on the BDAC-stabilized Au NBPs tips are displayed in Figures 6G-I. Notably, to increase the domain size of Cu_2‑x_Se on the tips, we not only add more SeO_2_ precursors but also use a higher concentration of BDAC surfactant, respectively. The only small Cu_2‑x_Se was overgrown on the tip of the BDAC-stabilized Au NBPs (Figure 6G) when only 25 μL of 1 mM SeO_2_ and 3 mM BDAC were used. On the other hand, the length of the semiconductor on the tips significantly increased when 50 and 75 μL of 1 mM SeO_2_ were added, respectively, to 5 and 7 mM BDAC (Figures 6H and 6I). The length difference of Cu_2‑x_Se by using 50 and 75 μL SeO_2_ was demonstrated in Figure S9. Cu_2‑x_Se domain size is 12 ± 2.5 nm when 50 μL SeO_2_ was used in 5 mM BDAC; otherwise, 22.0 ± 4.2 nm Cu_2‑x_Se was overgrown on the tip of Au NBPs with 75 μL SeO_2_ in 7 mM BDAC added. It is found that the domain size of Cu_2‑x_Se on the tip depends not only on the amount of SeO_2_ but also on the concentration of BDAC surfactant. This increase in length can potentially tune to the longitudinal LSPR of Au@Cu_2‑x_Se. As seen in Figure S10, the extinction UV–vis-NIR spectra gradually red-shifted with longer Cu_2‑x_Se on the tips. Additionally, the TEM image in Figure S10 confirms that these samples possess high quality and uniformity.

It is worth mentioning that these dual plasmonic Au@Cu_2‑x_Se hetero nanostructures exhibit high plasmonic stability (Figure S11), with well-controlled morphologies and Cu_2‑x_Se domain sizes, offering a versatile platform for advanced photocatalysis. They exhibit extraordinary tunability of extinction, broadening the light response from the UV–visible spectrum to the NIR region and enhancing hot carrier generation across the visible–NIR spectrum. This broad spectral response, combined with efficient hot carrier generation and strong photothermal conversion, leads to enhanced photocatalytic reactivity. Their application in photocatalytic nitrogen fixation will be presented in our forthcoming work.

## Conclusions

In summary, we have effectively developed a facile and general strategy for the site-selective overgrowth of Cu_2‑x_Se on the Au NBPs, which was obtained via a seed-mediated method with an aspect ratio of 2.64. The controllable synthesis of the high-quality monodisperse colloidal dual-plasmonic Au@Cu_2–x_Se heteronanostructures with a variety of morphologies was precisely manipulated on different positions on the Au NBPs surface by the Se-mediated two-step growth method with different capping agents such as CTAB, CTAB-PVP mixture, and BDAC-stabilized Au NBPs. Using CTAB as a surface stabilizer for Au NBPs, the overgrowth of Cu_2‑x_Se on one side of the Au NBPs’ waist creates an Au@Cu_2‑x_Se nanoUFO morphology. Alternatively, the island growth of Cu_2‑x_Se on both sides of Au NBPs, with the surface capped by a combination of CTAB and PVP, produced the Au@Cu_2‑x_Se nanoisland shape. Notably, the BDAC surfactant caused the Cu_2‑x_Se to be site-selectively deposited on the ends of Au NBPs, creating the Au@Cu_2‑x_Se nanospindle. Among them, Cu_2‑x_Se-tipped on Au NBPs was notable for having the largest longitudinal plasmon peak redshift in the visible and near-infrared ranges. This was caused by the combined oscillation of free holes in the semiconductor and hot electrons of the noble metal. More crucially, the Cu_2‑x_Se domain size was precisely adjusted, and subsequently tunable plasmonic properties were achieved by simply changing the amount of the SeO_2_ precursor. The results of this work provide further insight into the development of a regioselective growth technique for innovative dual plasmonic hetero nanostructures based on Au NBPs, supported by different capping agents. This will be the foundation for practical, extensive applications in solar energy, photovoltaics, photocatalysis, and biomedicine.

## Supplementary Material



## Notes

Experimental section including chemicals, growth of the involved Au NBPs, growth of Au NBPs@Cu_2‑x_Se nanoUFO, growth of Au NBPs@Cu_2‑x_Se nanoisland, growth of Au NBP@Cu_2‑x_Se nanospindle, characterization, and instrumentation. The supporting figures also include extinction spectra, TEM images, and size distributions. (PDF)
