# Supplementary material for: Precise Control over the Spatial Arrangement of Copper Selenide on Au Nanobipyramids by Site-Selective Growth for Dual Plasmonic Nanoarchitectures
Source: ACS Nanosci Au. 2025 Oct 20;5(6):461–8. doi: 10.1021/acsnanoscienceau.5c00102 (PMC12715625; doi:10.1021/acsnanoscienceau.5c00102)
Supplement: Supplementary file 1 [file ng5c00102_si_001.pdf]

Supporting Information

Precise Control over the Spatial Arrangement of Copper Selenide on Au Nanobipyramids by Site-selective Growth for Dual Plasmonic Nanoarchitectures

*Au Lac Nguyen and Hao Jing**

Department of Chemistry and Biochemistry, George Mason University, Fairfax, Virginia 22030, USA

* To whom correspondence should be addressed.
Email: hjing2@gmu.edu; Phone: 1-703-993-5221; Fax: 1-703-993-1040.

Materials. Hydrogen tetrachloroaurate (III) trihydrate($HAuCl_4.3H_2O$), 99.99%), hexadecyltrimethylammonium chloride (CTAC, >95.0%), sodium citrate tribasic dihydrate (99.0%), sodium borohydride ($NaBH_4$, 99.99%), hexadecyltrimethylammonium bromide (CTAB, >98.0%), L-ascorbic acid (AA ≥ 99.0%), silver nitrate ($AgNO_3$), hydrochloric acid (HCl) distilled ethanol (C_2H_5OH, >99.99%), selenium dioxide (≥ 99.9%), copper (II) sulfate (99.99%), polyvinylpyrrolidone (PVP, MW= 55,000), benzyl dimethyl hexadecyl ammonium chloride (BDAC), were all purchased from Sigma-Aldrich. All chemicals were used as received without further purification. Ultra-pure deionized water (MilliQ, 18.2 MΩ.cm at 25 ^{o}C) was used in the experiment.

Growth of Gold Nano Bipyramids

In this work, gold nano Bipyramids **(**Au NBPs) were synthesized using seed-mediated growth processes as described in previous works. Briefly, $HAuCl_4$ (5 mL, 0.25 mM) was quickly reduced by freshly prepared ice-cold $NaBH_4$ (0.125 mL, 25 mM) in an aqueous solution containing CTAC (2.5 mL, 200 mM) and 1 $CiNa_3$ (2.375 mL, 10 mM) under vigorous stirring at ambient temperature for 2 minutes. The color of the gold seed solution changed from a faint yellow to a brownish hue. The mixture was then placed in an oil bath at 80 ^{0}C and stirred at 300 rpm. After aging for 2 hours, the resultant gold seed solution changed to a translucent red color and was then removed from the oil bath and stored at room temperature. Next, 125 µL of seed solution was then quickly injected into the growth solution, which contained 10 mL of 100 mM CTAB, 0.5 mL of 10 mM $HAuCl_4$, 0.1 mL of 100 mM $AgNO_3$, 0.2 mL of 100 mM HCl, and 0.08 mL of 100 mM AA. The mixture was gently stirred for 2 minutes and then left undisturbed in an oil batch at 30°C for 2 hours. The Au NBPs are obtained by washing the colloid twice at 6000 rpm for 6 minutes with ultrapure water and then was redispersed in 10 mL of 5 mM CTAB.

Growth of Au@$Cu_{2-x}Se$

Dual plasmonic Au@$Cu_{2-x}Se$ hetero nanostructures with different morphologies were synthesized through a two-step process: 1) surface modification of Au NBPs and 2) the Selenium-Mediated approach.

Au@$Cu_{2-x}Se$ nanoUFO hetero-nanostructure

Typically, the first step in synthesizing the Au@$Cu_{2-x}Se$ nanoUFO shape involved adding 300 µL of Au NBP solution to 1.2 mL of 5 mM CTAB and stirring for 5 minutes. The second step involves several sequential sub-steps. First, 100 µL of 0.1 M AA aqueous solution was added to the Au NBP diluted solution at 40 ^{0}C. Subsequently, 50 µL of 1 mM SeO_2 aqueous solution was added dropwise using a syringe pump with an injection rate of 80 µL/min, while stirring at 1200 rpm. After the injection had been completed for 5 minutes, the mixture was cooled to room temperature, and 10 µL of 0.2 M $CuSO_4$ was injected simultaneously with 100 µL of 0.1 M AA aqueous solution into the mixture at room temperature. The mixture was then left under vigorous stirring at 1200 rpm for 30 minutes. The resulting products were purified through centrifugation at 6000 rpm for 3 minutes and redispersed in water for future use. Furthermore, the varying shell thickness of $Cu_{2-x}Se$ samples was also successfully synthesized by adjusting the amount of 1 mM SeO_2 to 25 and 100 µL, using the same protocol.

Au@$Cu_{2-x}Se$ Nanoisland hetero-nanostructure

The first step in synthesizing Au@$Cu_{2-x}Se$ nanoislands involved surface treatment using binary surfactants. Briefly, 300 µL of the as-prepared Au NBP solution was centrifuged twice at 6000 rpm for 5 minutes using ultrapure water. The residue was redispersed in 1.5 mL of 3 mM CTAB.

The solution was stirred for 5 minutes before 100 μL of a 50 mg/mL PVP solution was subsequently added and gently stirred for an additional 20 minutes. The second step was the same as the UFO synthesis protocol. Furthermore, the varying shell thickness of $Cu_{2-x}Se$ samples was also successfully synthesized by adjusting the amount of 1 mM SeO_2 to 25 and 100 μL, using the same protocol.

Au@$Cu_{2-x}Se$ nanospindle hetero-nanostructure

In typical synthesis, as-prepared Au NBPs were washed twice to remove the excess surfactant capping on the particle surface. After that, the residue was redispersed into 1.5 mL of 5 mM BDAC. Then, 10 μL of 1 mM $AgNO_3$ solution was added to the Au NBP solution under gentle shaking for 1 minute, followed by the addition of 100 μL of a 0.05 mM AA aqueous solution to the mixture for an additional 2 minutes under gentle stirring. The mixture was then left undisturbed in an oil bath at 70 ^{0}C for 20 minutes. The second step of the $Cu_{2-x}Se$ coating synthesis was slightly different from the previous protocol. Specifically, 80 μL of 0.05 M AA aqueous solution was added to the Au NBP diluted solution at 40 °C. Subsequently, 50 μL of 1 mM SeO_2 aqueous solution was added dropwise using a syringe pump with an injection rate of 80 μL/min, while stirring at 1200 rpm. After the injection had been completed for 5 minutes, the mixture was cooled to room temperature, and 10 μL of 0.2 M $CuSO_4$ was injected simultaneously with 80 μL of 0.05 M AA aqueous solution into the mixture at room temperature under vigorous stirring at 1200 rpm for 30 minutes. The resulting products were purified through centrifugation at 6000 rpm for 3 minutes and redispersed in water for future use. Furthermore, the varying shell thickness of $Cu_{2-x}Se$ samples was also successfully synthesized by adjusting the amount of 1 mM SeO_2 to 25 and 70 μL with different concentrations of BDAC to 3 and 7 mM, respectively, using the same protocol.

Characterization and Instrumentation.

The optical extinction spectra of colloidal gold seeds and Au NBPs were recorded using a Shimadzu UV-2600 spectrophotometer at ambient temperature, equipped with quartz cuvettes of 1.7 mL volume. The crystal structures were determined using an X-ray diffractometer (Rigaku, Japan) with Cu Kα radiation ($\lambda = 1.5406$ Å) at room temperature, with a scattering angle 2θ ranging from 20° to 80° for 60 minutes. The ligand binding between capping agents and Au@$Cu_{2-x}Se$ surface was determined by Ocean Optics Raman spectrometer. The size and morphological structure of the obtained Au NBPs were observed using a transmission electron microscope (TEM, JEOL JEM-1400Flash) operating at 120KV. HRTEM, HAADF-STEM imaging, and EDX elemental mapping of these samples were cast on the Ni grids and carried out using a Thermo Fisher Scientific, probe-corrected Themis Z, which was operated at 200 kV in STEM mode with bright-field and high-angle annular dark-field detectors. The EDX was performed using a 4-segment super X detector. Probe current 200pA.

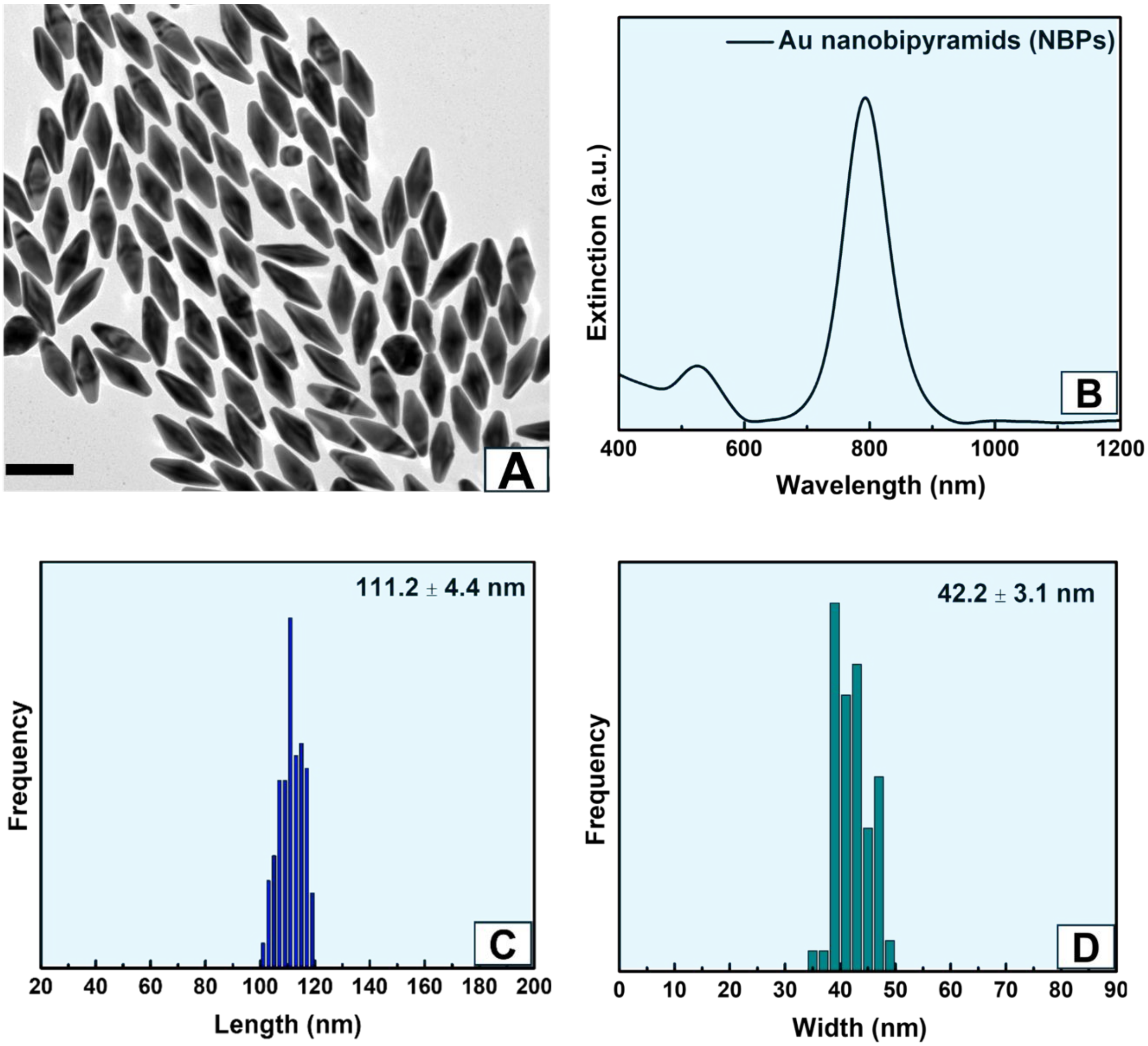

Figure S1. As-prepared Au NBPs was synthesized by using binary surfactants. A) TEM images of monodisperse synthesized Au NBPs, B) Extinction spectra of as-prepared Au NBPs, C and D length and width distribution of as-prepared Au NBPs.

The morphology of Au@$Cu_{2-x}Se$ is strongly influenced by both the halide ions (Br^- vs. Cl^-) and the cationic surfactant headgroups (CTA^+ vs. BDA^+). Bromide ions from CTAB are known to adsorb preferentially onto specific Au facets, thereby suppressing growth on those surfaces and directing site-selective overgrowth into UFO-like structures. Whereas chloride ions from BDAC exhibit weaker binding, leading to different morphologies. Beyond the halide effect, the nature of the cationic headgroup (CTA^+ vs. BDA^+) also modulates micelle packing and surface interactions, further contributing to the regioselective overgrowth of $Cu_{2-x}Se$ on Au NBPs. To systematically probe these factors, we carried out four control experiments using CTAB and BDAC in the

presence and absence of Ag^+, with corresponding TEM images provided in the Supporting Information.

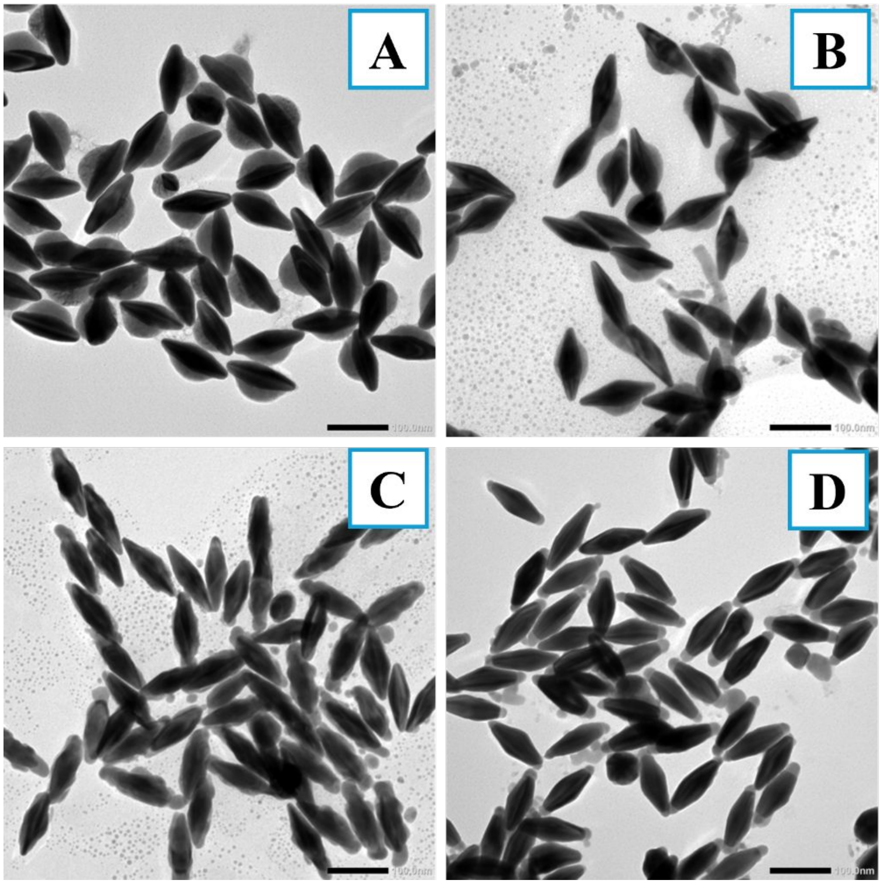

Figure S2. TEM images of Au@Cu_{2-x}Se synthesized using different surfactants (CTAB or BDAC) with and without Ag^+. (A) CTAB without Ag^+, (B) BDAC without Ag^+, (C) CTAB with Ag^+, and (D) BDAC with Ag^+. The scale bar is 100 nm.

The results indicate that Au@Cu_{2-x}Se heterostructures synthesized with different surfactants in the absence of Ag^+ exhibited similar morphologies, yielding predominantly UFO-like structures with high uniformity for both CTAB and BDAC (Figure S2. A, B). In contrast, when Ag^+ was introduced, distinct morphologies were obtained: CTAB-directed synthesis produced a core–shell structure, whereas BDAC led to the formation of well-defined spindle-like architectures (Figure S2. C, D). These findings demonstrate the synergistic effects of halide ions, surfactant headgroups, and Ag^+ in directing site-selective Cu_{2-x}Se overgrowth on Au NBPs.

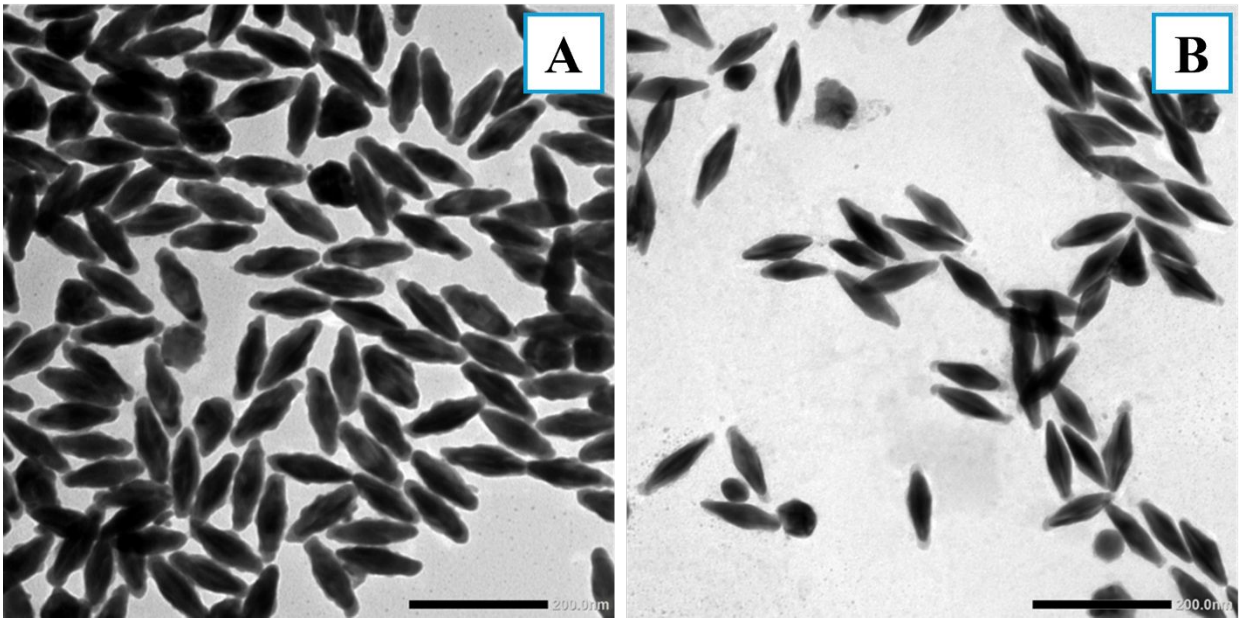

Figure S3. TEM images of $Au@Cu_{2-x}Se$ spindles with BDAC concentrations of (A) 1 mM and (B) 10 mM. The scale bar: 200 nm.

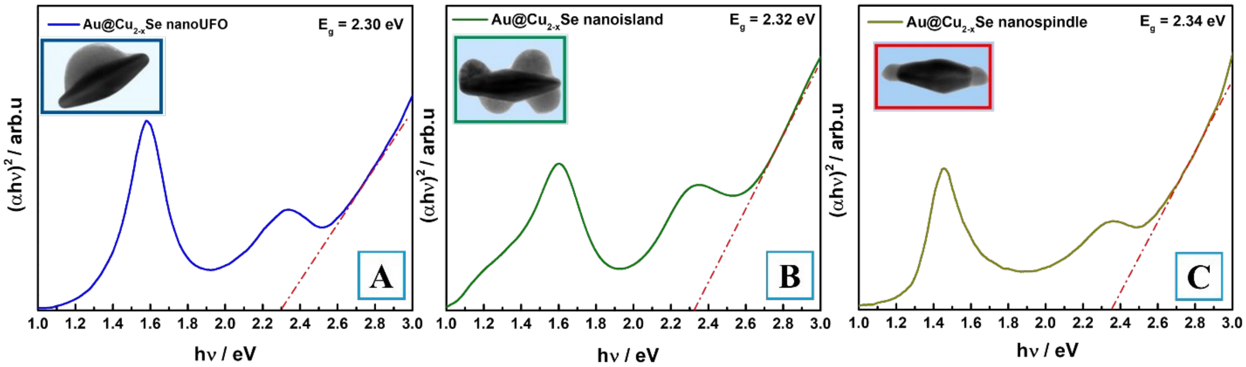

Figure S4. The band gap energy of three different $Au@Cu_{2-x}Se$ samples A) nanoUFO, B) nanoisland, C) nanospindle.

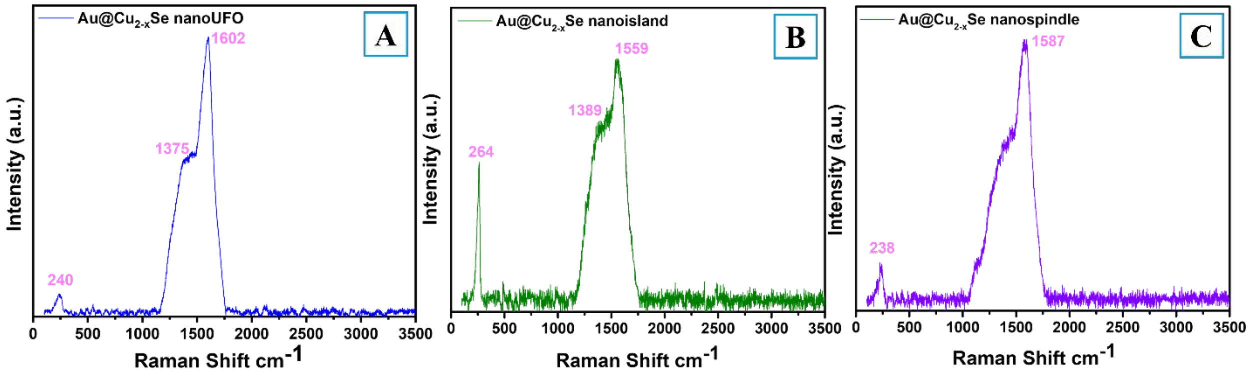

Figure S5. Raman spectra of Au@$Cu_{2-x}Se$ hetero nano structures functionalized with different surfactants: (A) nanoUFO sample stabilized with CTAB (B) nanoisland sample obtained from a CTAB–PVP mixture, (C) nanospindle sample stabilized with BDAC.

The Raman spectra of three Au@$Cu_{2-x}Se$ samples consistently reveal a Cu–Se stretching mode at ≈238–264 cm^{-1}, confirming the formation of crystalline copper selenide domains. For the UFO sample, a CH_2 band at ≈1375 cm^{-1} indicates ordered CTAB alkyl chains, while the ≈1602 cm^{-1} feature reflects perturbed headgroup/Br^- interactions with the Au–$Cu_{2-x}Se$ surface. These results suggest that CTAB stabilizes the hybrid by binding through Br^- adsorption to surface metal sites, with CTA^+ headgroups and alkyl tails forming an ordered shell. In the Island sample, bands at approximately 1389 and 1559 cm^{-1} arise from the organic shell. CH_2 deformations again signal ordered CTAB alkyl chains, while the downshifted and broadened carbonyl band indicates a strong perturbation of PVP. The red shift of the PVP carbonyl suggests ion–dipole or coordination interactions with Br^--modified Au NBP surfaces. Together, these features support a cooperative stabilization mechanism in which Br^- anchors the surface, CTAB organizes laterally, and the carbonyl groups of PVP direct facet-selective $Cu_{2-x}Se$ growth. The Spindle sample exhibits strong CH_2 modes and a benzyl C=C band at 1587 cm^{-1}, confirming the presence of densely packed BDAC molecules on the surface.

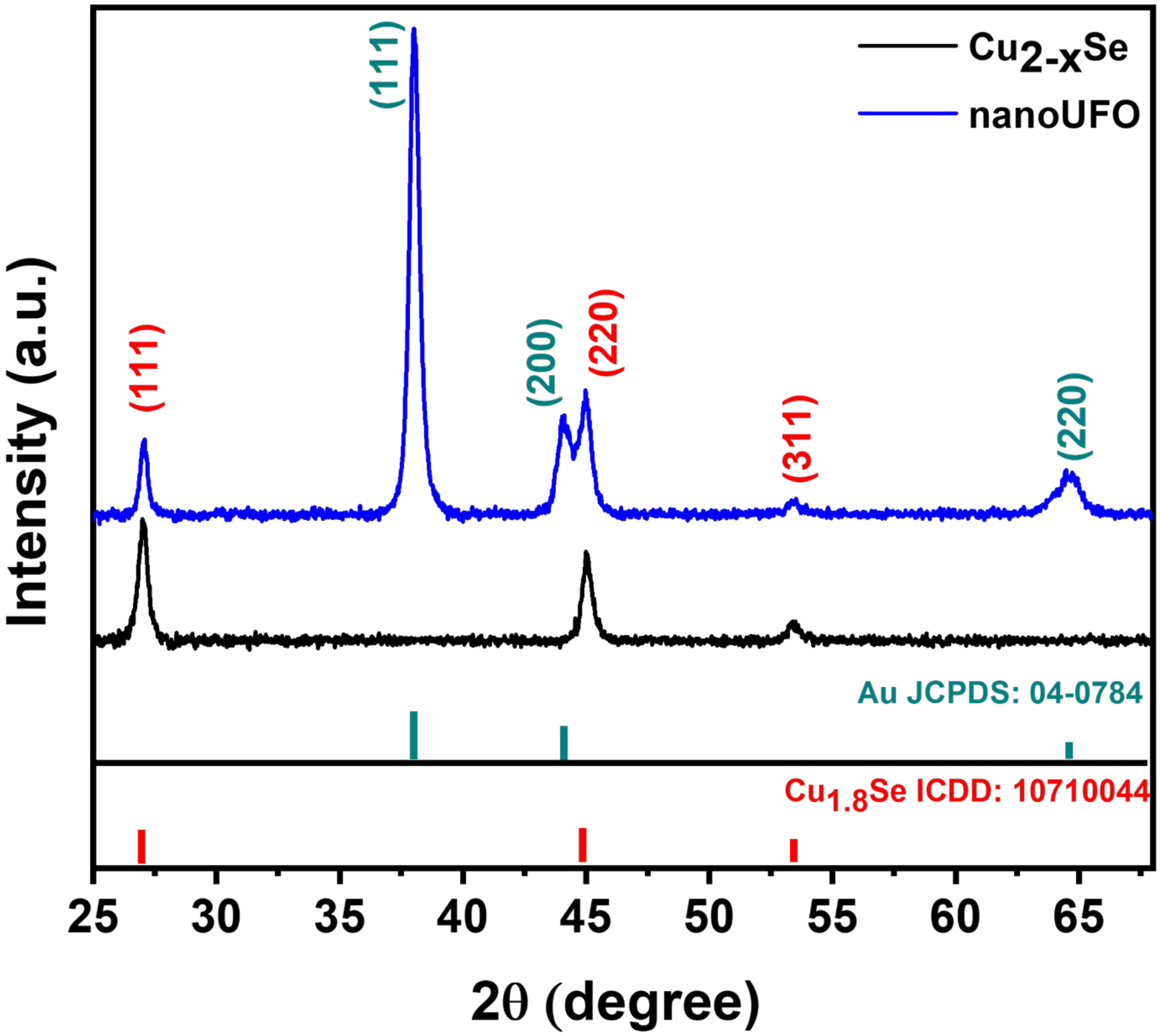

Figure S6. Powder XRD patterns of Au@$Cu_{2-x}Se$ and $Cu_{2-x}Se$ showed the simultaneous existence of gold and copper selenide crystal phases. XRD analysis was carried out to further investigate the structural characteristics of the Au@$Cu_{2-x}Se$ and $Cu_{2-x}Se$ samples. As shown in the diffraction patterns, the Au@$Cu_{2-x}Se$ heterostructure exhibits well-defined peaks, confirming its high crystallinity. The characteristic reflections at 2θ = 27.04°, 44.94°, and 53.48° can be indexed to the crystalline $Cu_{2-x}Se$ phase, while additional peaks at 2θ = 37.98°, 43.98°, 64.66°, and 77.34° are assigned to the face-centered cubic structure of metallic Au, verifying the coexistence of both components in the hybrid material.

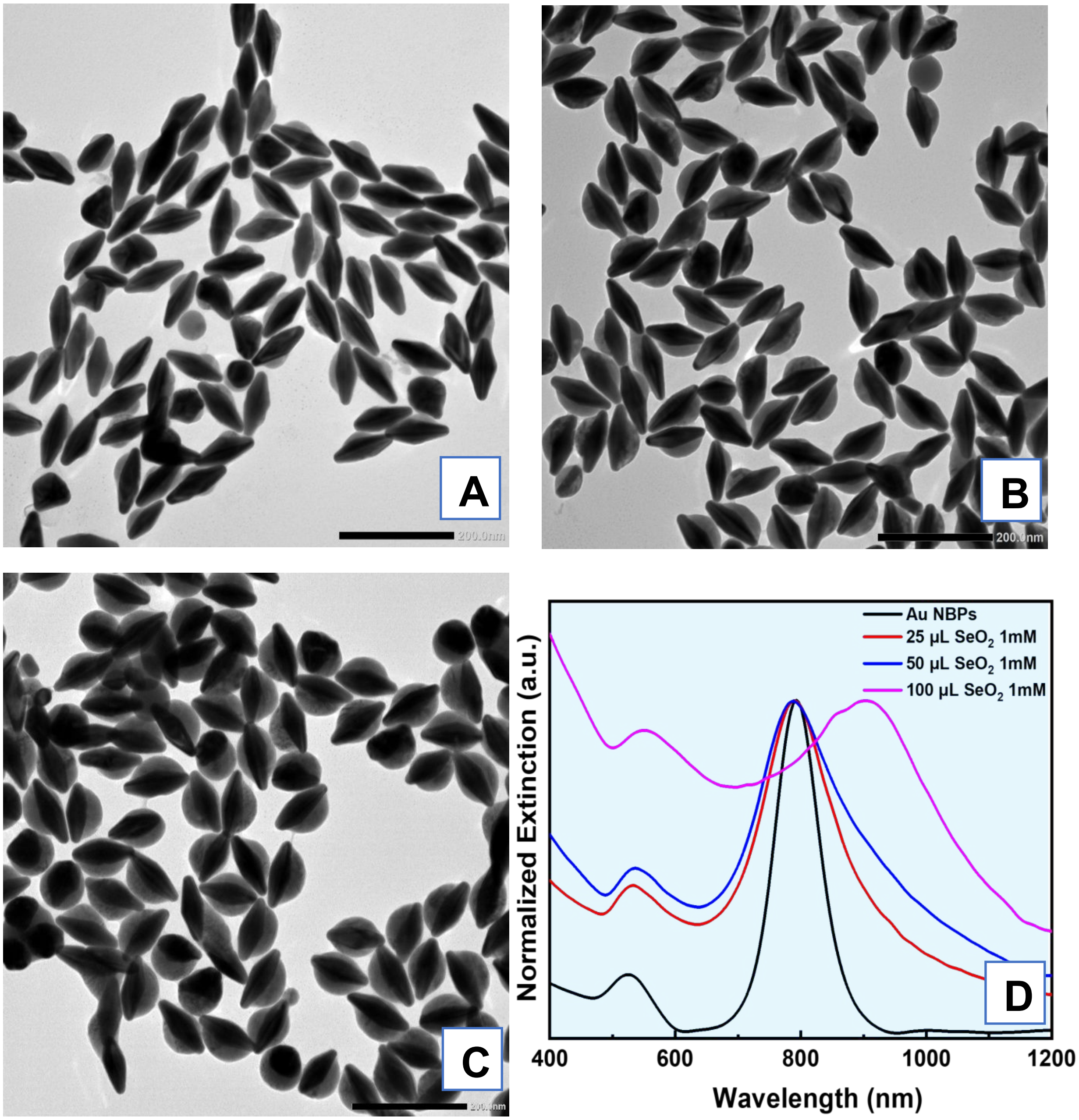

Figure S7. The TEM images of three Au@$Cu_{2-x}Se$ nanoUFO hetero-nanostructure samples with various $Cu_{2-x}Se$ thicknesses. A) 25 µL of 1 mM SeO_2, B) 50 µL of 1 mM SeO_2, C) 100 µL of 1 mM SeO_2, D) Extinction spectra of three samples with different thicknesses and as-prepared Au NBP. The Scale bar is 200 nm.

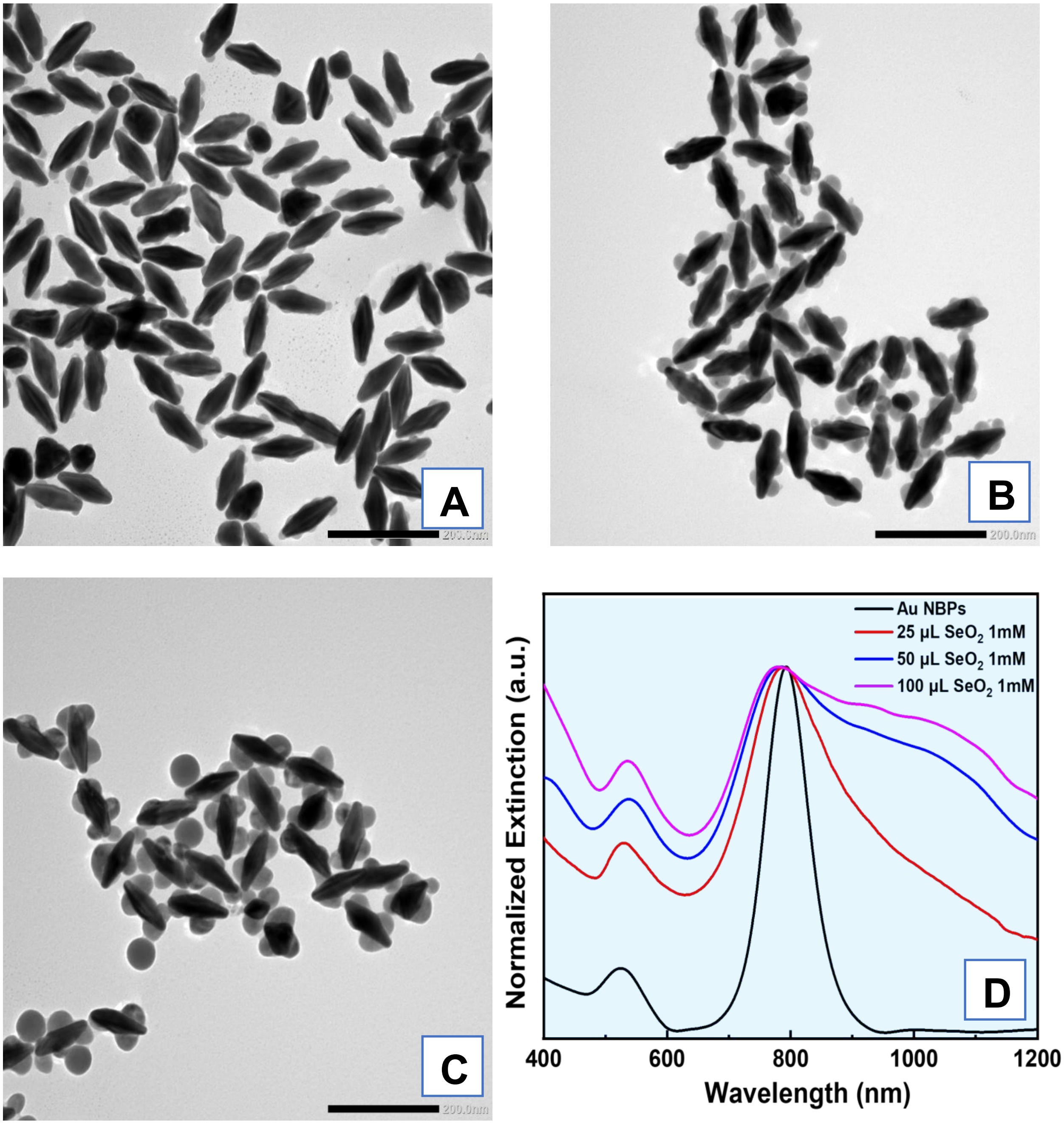

Figure S8. The TEM images of three Au@$Cu_{2-x}Se$ nanoisland hetero-nanostructure samples with various $Cu_{2-x}Se$ thicknesses. A) 25 μL of 1 mM SeO_2, B) 50 μL of 1 mM SeO_2, C) 100 μL of 1 mM SeO_2, D) Extinction spectra of three samples with different thicknesses and as-prepared Au NBP. The Scale bar is 200 nm.

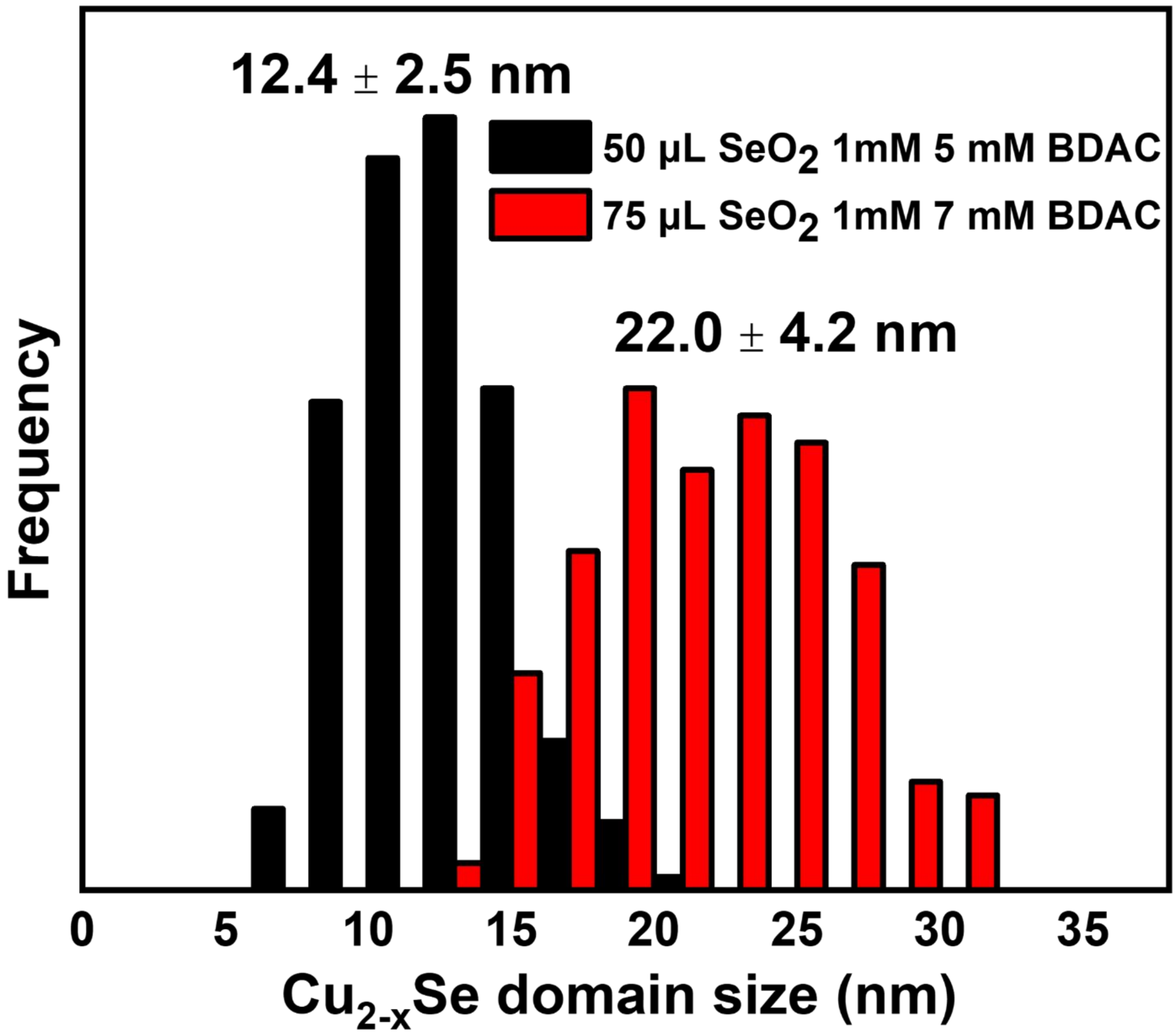

Figure S9. The distribution of the length of $Cu_{2-x}Se$ on the BDAC-stabilized Au NBP tips. The red column represents a sample with 75 μL SeO_2 in BDAC 7 mM, and the black column represents a sample with 50 μL SeO_2 in BDAC 5 mM. Size distributions are obtained by counting 150 particles of each sample.

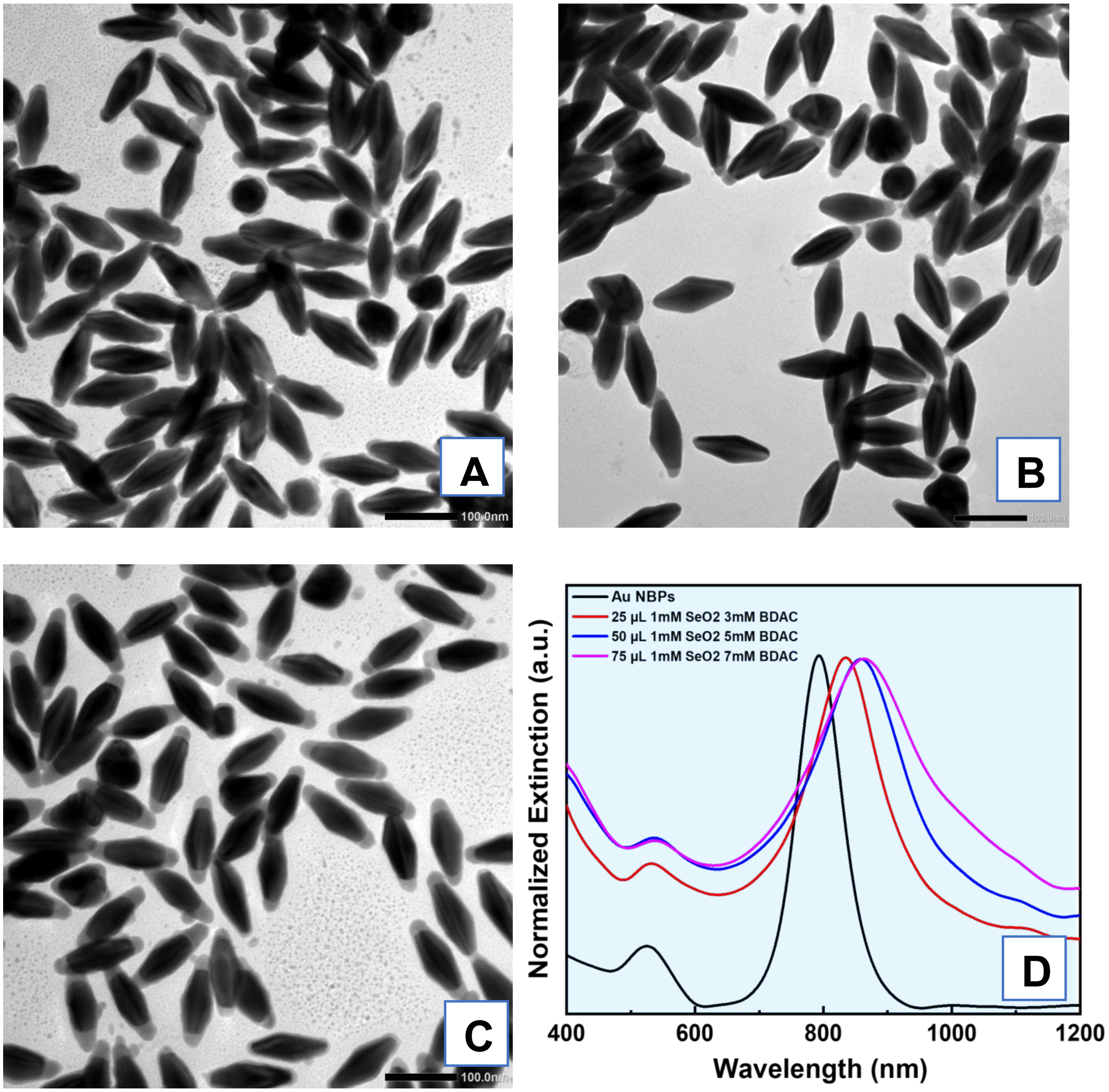

Figure S10. The TEM images of three Au@$Cu_{2-x}Se$ nanospindle hetero-nanostructure samples with various $Cu_{2-x}Se$ domain sizes. A) 25 µL of 1 mM SeO_2 in 3 mM BDAC, B) 50 µL of 1 mM SeO_2 in 5 mM BDAC, C) 75 µL of 1 mM SeO_2 in 7 mM BDAC, D) Extinction spectra of three samples with different thickness and as-prepared Au NBP. The Scale bar is 100 nm.

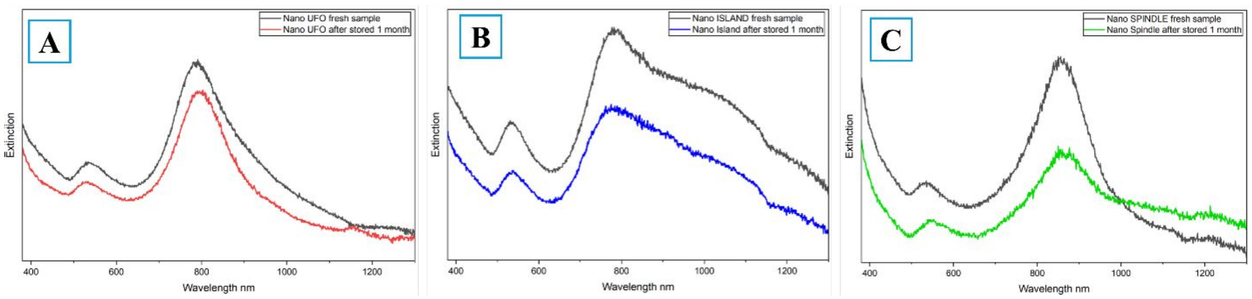

Figure S11. Extinction Spectra of Au@$Cu_{2-x}Se$ varying morphologies right after synthesis and after being stored for one months. A) Au@$Cu_{2-x}Se$ nano UFO, B) Au@$Cu_{2-x}Se$ nano island, C) Au@$Cu_{2-x}Se$ nano spindle.

To evaluate the plasmonic stability of the Au@$Cu_{2-x}Se$ samples, we re-examined their optical properties after storing them under ambient conditions and in the dark for one month. The extinction spectra confirm that the samples remain highly stable over this period. The LSPR peaks are preserved at the same spectral positions, indicating that no significant structural or compositional changes occurred. A slight decrease in peak intensity was observed; however, this is most likely due to minor particle loss during sample handling and measurement, rather than to the intrinsic instability of the nanostructures. These results demonstrate the excellent long-term plasmonic stability of the Au@$Cu_{2-x}Se$ heterostructures.
